# Cross-diagnostic determinants of cognitive functioning: the muscarinic cholinergic receptor as a model system

**DOI:** 10.1038/s41398-023-02400-x

**Published:** 2023-03-27

**Authors:** Sara E. Jones, Philip D. Harvey

**Affiliations:** 1 Department of Psychiatry, University of Miami Miller School of Medicine, Miami, FL USA; 2 Department of Psychiatry, Washington University School of Medicine, St Louis, MO USA; 3 Research Service, Bruce W. Carter VA Medical Center, Miami, FL USA

**Keywords:** Schizophrenia, Predictive markers

## Abstract

Cognitive impairment is a predictor of disability across different neuropsychiatric conditions, and cognitive abilities are also strongly related to educational attainment and indices of life success in the general population. Previous attempts at drug development for cognitive enhancement have commonly attempted to remedy defects in transmitters systems putatively associated with the conditions of interest such as the glutamate system in schizophrenia. Recent studies of the genomics of cognitive performance have suggested influences that are common in the general population and in different neuropsychiatric conditions. Thus, it seems possible that transmitter systems that are implicated for cognition across neuropsychiatric conditions and the general population would be a viable treatment target. We review the scientific data on cognition and the muscarinic cholinergic receptor system (M1 and M4) across different diagnoses, in aging, and in the general population. We suggest that there is evidence suggesting potential beneficial impacts of stimulation of critical muscarinic receptors for the enhancement of cognition in a broad manner, as well as the treatment of psychotic symptoms. Recent developments make stimulation of the M1 receptor more tolerable, and we identify the potential benefits of M1 and M4 receptor stimulation as a trans-diagnostic treatment model.

## Introduction

In 2016, schizophrenia was determined to be the 12^th^ most disabling disorder globally in terms of healthy years of life lost [1]. Schizophrenia poses tremendous societal costs, including direct costs for treatment and drugs, but also indirect costs such as lost productivity, mortality, family impact, and criminal justice system costs; while affecting 0.7% of the population, schizophrenia typically accounts for 1.5–3% of all nations’ total health care expenditures [2]. Previous studies have reported that, as schizophrenia significantly impairs the ability of patients to perform social, vocational, and everyday living tasks [3], more than 70% of people with schizophrenia are challenged with living independently and finding employment [4, 5]. Despite the significant healthcare and societal resources dedicated to schizophrenia, adult-onset schizophrenia has only a 13.5% recovery rate [6], with recovery rates even lower for childhood onset.

While schizophrenia is defined diagnostically by positive symptoms such as hallucinations and delusions, cognitive deficits are much greater determinants of long-term disability and poor functional prognosis [7, 8]. Despite the considerable impact of neurocognitive deficits on function, current treatments for schizophrenia do not adequately address these dimensions. Given the magnitude of disability caused by the cognitive impacts of schizophrenia, treatments that may address these domains should be pursued. Accordingly, other targets beyond the dopamine antagonism used to treat psychosis seem critical to pursue. There are multiple other transmitter systems, as well as elements of brain structure and function, that may determine cognitive and functional impairments. Efforts to target these systems have also not been successful, for reasons addressed by the authors of those earlier reviews. One reason for concern, as we describe below, is that pharmacological treatment efforts aimed at cognition in schizophrenia have typically been aimed at neurotransmitter systems that have been implicated as having abnormalities in schizophrenia, generally related to the origin of psychotic symptoms. However, considerable genomic evidence from very large-scale Genome Wide Association studies (GWAS) has suggested that cognitive deficits in schizophrenia (and other serious mental illnesses) share genomic variance with polygenic scores (PS) for cognition (as well as intelligence and educational attainment) in the general population, possibly more than with PS for schizophrenia [9]. Also, PS for cognition in other serious mental illnesses, such as bipolar illness, shares variance with PS for cognition in the general population and in schizophrenia [10]. Thus, impairments in cognition in serious mental illness (SMI) may be best conceptualized as originating from factors that are operative in the general population in addition to disease specific processes across neuropsychiatric conditions.

These findings implicate several possible new strategies for cognitive enhancement on a pharmacological level in serious mental illness. If the genomics of cognition is similar in SMI and the general population, why should pharmacological treatment targets be diagnosis specific? Cognitive impairment seems to be more considerably more similar across DSM-based entities than symptom severity, questioning the need to consider cognitive impairments differentially across diagnostic entities. Cognitive impairments are modestly correlated with symptom severity on both cross-sectional and longitudinal bases in schizophrenia and bipolar disorder, as evidenced by the persistence of cognitive impairments in individuals with both diagnoses who are clinically stable [e.g [11, 12]]. The correlation between symptomatic status in bipolar disorder and cognition appears modest based on multiple studies of impairments in euthymic participants with bipolar illness and their generally unaffected relatives [13].

Finally, large scale studies of the similarity of cognitive deficits in these two conditions are beginning to appear. In the largest study to date focusing on this issue (*n* = 10,160), we [14] found that analyses of the factor structure of cognition across diagnoses manifested the best fit to the data when characterized as a single domain. These findings were substantiated by the results of genomic analyses like those described above [15], which found that a common latent trait for cognition across the two diagnoses was related to previous PS for cognition, intelligence, and educational attainment in general population samples [16–18]. There was no evidence for diagnosis specific profiles of cognitive impairment or differential genomic correlates of cognitive profiles across diagnoses, with greater differences in PS correlates based on racial status than on diagnosis. Multiple studies have suggested that cognition in schizophrenia is multifactorial, but the larger the study, the more likely that a unifactorial solution will be found. For example, in a large-scale study of cognition in participants with schizophrenia, bipolar disorder, and their unaffected relatives, as well as healthy controls (*n* = 2066) [19], cognitive performance measured with the Brief Assessment of Cognition in Schizophrenia (BACS) [20] was best defined as a unitary latent trait across all the different participant groups. A unifactorial solution based on neuropsychological tests does not mean that cognition has a single origin, but the similarity of findings of cognitive deficits on a cross diagnostic basis (using the tests that are strongly correlated with functional disability) suggests that cross-diagnostic therapeutic efforts may be equally as reasonable as diagnosis specific studies.

As a result, it may be important to consider treatment targets that may be relevant to cognition across psychiatric and neuropsychiatric conditions, as well as in the general population. There are several reasons that this may be important, as reviewed above. First, the results of genomic studies and large-scale assessment-based studies suggest that the differences in performance across neuropsychiatric conditions are generally limited to differences in the severity of impairment that could be related to differences in premorbid functioning. Cognitive performance across diagnoses has similar correlational structures, and similar genomic correlates. Second, it is widely understood that symptomatic variables (psychosis, current mood state, negative symptoms) in schizophrenia and bipolar disorder (and to an extent in major depression [21]) are minimally correlated with concurrent cognitive impairment. Thus, for the rest of this paper we will review the importance of a neurotransmitter system where evidence has suggested that there is potential cross-diagnostic importance in cognition: the muscarinic cholinergic receptor system, with discussion of receptor subtypes and comparison with the nicotinic cholinergic system.

Aside from SMI, there are other diagnosed conditions where cognitive impairments are central features of the illness, including Alzheimer’s disease (AD) and related amnestic mild cognitive impairment (aMCI). Interesting and convincing evidence that AD was associated with reduced muscarinic cholinergic functioning [22] lead to the pursuit of treatments targeting this mechanism. Both muscarinic M1 agonists [23] and acetylcholinesterase inhibitors (ACHI) [24–26] were explored beginning two or more decades ago as treatments for cognitive impairments in AD and aMCI. These same treatments were also explored in healthy aging populations, albeit in small sample studies [27], focusing on improvements in episodic memory. Thus, it could be argued that the muscarinic cholinergic transmitter systems are relevant to cognitive functioning across diagnoses and that interventions aimed at improving the functioning of the system can lead to benefits in the domains targeted.

There is considerable evidence of the importance of the muscarinic cholinergic system in schizophrenia [28]. Muscarinic agonists were discovered to have antipsychotic-like activity in animal models [29], although it is not clear if this is due to M1 or M4 agonist effects [30]. Cognitive functioning in schizophrenia, like in AD, was reported to be improved by treatment with the M1/M4 agonist xanomeline [31]. Thus, these data suggest that manipulations affecting other neurotransmitter systems, not typically targeted in the treatment of schizophrenia, may exert beneficial effects on cognition across apparently very different (neurodegenerative vs. not; aging related vs. not) classes of neuropsychiatric conditions.

This is not a new concept. When amphetamine treatments were introduced for attention deficit disorders, younger and older healthy comparison participants commonly manifested the same degree of improvements in performance observed in the ADHD treatment samples [32]. Evidence from multiple studies has suggested that stimulant administration, at the correct doses, even in unimpaired samples, leads to improvements in test performance [33]. Thus, a baseline deficit in monoamine functioning is not required to detect an improvement in cognitive performance after dosing with an augmenting agent. Further, cognitive test performance [34] and response to cognitive training [35] are improved in participants with schizophrenia when treated with stimulants, despite concurrent treatment with antipsychotic medications. Thus, it is entirely possible that cross-diagnostic facilitation of cognition can be achieved with a single treatment.

Xanomeline, an M1/M4 muscarinic receptor agonist, has sparked renewed interest in its utility as a possible treatment for schizophrenia. Compared to dopamine antagonists, which rely on the dopamine D_2_ receptor blockade and preferentially treat positive symptoms, muscarinic agonists such as xanomeline may offer a different level of transmitter interaction in terms of reduction of psychosis. They also offer potential to address cognitive symptomology in both AD and schizophrenia [36]. While xanomeline was initially investigated in the 1990s, trials were discontinued due to intolerable peripheral cholinergic side effects. In a new formulation, xanomeline is combined with trospium chloride, a pan-muscarinic receptor antagonist that does not cross the blood-brain barrier, thus limiting the effects of xanomeline to the central nervous system (CNS) and potentially improving its side effect profile [37]. We will next examine the neuroscience underpinnings of this general treatment strategy and evaluate the role of agents affecting both M1 receptors and M4 receptors in the treatment of psychosis and other neuropsychiatric pathologies.

## Cholinergic systems

Acetylcholine was the first neurotransmitter discovered. In the CNS, the cholinergic system has been implicated in numerous processes including sensory perception, motor function, cognitive processing, memory, attention, mood, and psychosis [38, 39]. Of note, the cholinergic system is believed to be the primary regulator of learning, memory, and attention functions [40], in both human [41] and animal models [42]. M1 receptor blockade produces reliable, dose and time course dependent, cognitive impairments in healthy people, younger and older [43], which can be reversed by agents that stimulate the cholinergic system [44]. Drugs that enhance cholinergic functioning produce cognitive improvements in healthy people who were selected for the absence of any cognitive impairments [27]. Accordingly, degeneration of the central cholinergic system is considered a driving force of Alzheimer’s dementia (AD) and even normal age-related memory changes [45]. Similarly, cholinergic functioning has been implicated in the pathophysiology of reduced cognition in schizophrenia, as studies have shown that neurocognition decreases in patients with schizophrenia after exposure to anticholinergic medications [46–48]. This association is particularly problematic given that some of the first and second-generation antipsychotics, the standard treatment for schizophrenia, have anticholinergic properties [49]. More imoprtantly, some of these medications cause extra-pyramidal symptoms, which are themselves treated with anticholinergic medications. Thus, current treatments for schizophrenia may worsen neurocognitive dysfunction through increasing the need for anticholinergic treatments.

In the periphery, the cholinergic system is responsible for the parasympathetic nervous system and regulates various functions including gastrointestinal motility, sweat production, smooth muscle activity, and heart rate. Accordingly, pharmaceuticals that act as cholinergic agonists in the periphery are prone to cause related side effects, including diarrhea, excessive sweating and salivation, and bradycardia. While muscarinic agonists such as xanomeline have shown promise in improving cognition through CNS cholinergic activation, dosing has ultimately been limited due to these peripheral side effects [50].

The cholinergic system contains two families of acetylcholine receptors: muscarinic receptors and nicotinic receptors. Nicotinic receptors are ligand-gated ion channels composed of 5 subunits; when acetylcholine binds to the receptor, an influx of sodium ions causes a rapid response [51]. Interest in development of nicotinic agents was spurred by the high prevalence of smoking on the part of people with schizophrenia and suggestions of alterations of genomic alterations associated with nicotinic cholinergic receptors [52, 53]. Proof of concept studies showed that nicotinic alpha-7 agonists could exert some benefits on cognition in schizophrenia [54], but a phase 2 trial was unsuccessful [55]. There appear to be considerable differences across these compounds in their receptor affinities and binding durations and other compounds such as EVP-6124 (encenicline), which targets the alpha-7 nicotinic receptor and showed preliminary promise in improving cognitive function in patients with schizophrenia and AD [56, 57], but did not separate from placebo in a phase 3 clinical trial. The development program was stopped and possible beneficial features of encenicline on psychotic symptoms coming from basic science studies have not been tested in humans [58]. Another alpha-7 nicotinic partial agonist was tested in phase 2B studies and did not separate from placebo in either smoking or nonsmoking participants with schizophrenia [59, 60] with the fact remaining that no alpha-7 nicotinic receptor has led to benefits on cognition that separated from placebo in an FDA-endorsed phase 3 clinical trial.

In contrast to nicotinic receptors, muscarinic receptors are G-protein coupled receptors which impact intracellular messengers and thus have a less rapid onset but longer lasting effect on cholinergic transmission than stimulation of nicotinic receptors [61]. 5 different subtypes of muscarinic receptors have been identified, and all have been found in the CNS at varying concentrations [62, 63]. Studies conducted with muscarinic receptor knockout mice suggest that the M1 receptor is primarily involved in neurotransmission for learning and memory, while the M4 receptor is largely involved in dopamine regulation [64]. M2 and M3 receptors, while sparingly present in the CNS, appear to primarily control the peripheral parasympathetic nervous system, with large concentrations in the heart and gastrointestinal smooth muscle [65]. Finally, M5 receptors in the periphery appear responsible for dilation of cerebral arteries, while their role in the CNS is less clear [62]. Accordingly, M1 and M4 have become targets for the treatment of psychosis and cognition in schizophrenia and Alzheimer’s disease; however, concurrent activation of M2 and M3 must be avoided to prevent peripheral side effects. Of note, while M1 is primarily expressed in the CNS, it is also expressed in lesser quantities in the periphery, which means that M1 activation may also result in peripheral effects.

### M4 receptors and dopamine regulation

M4 is expressed preferentially in the substantia nigra, with lesser expression in the cortex and hippocampus [63]. M4 knockout mice exhibit hyperdopaminergic activity [65, 66], suggesting that M4 is responsible for downregulating dopamine release. It has been proposed that activation of the M4 muscarinic receptor inhibits dopamine release through cholinergic projections to the substantia nigra [67, 68]. This mechanism is supported by findings that M4 agonists can reverse D1 agonist-induced hyperlocomotion [69]. That same study reported that M4 agonism may concurrently enhance dopamine release in the hippocampus and cortex, thus increasing neurocognitive functioning. Importantly, while M4 agonists prevent D1 activation in the substantia nigra, they do not inhibit D1 function in the cortex or hippocampus, suggesting that M4 agonists may be capable of selectively inhibiting dopamine release in a way that dopamine antagonists, or antipsychotics, are not.

Prior imaging studies have found that patients with schizophrenia have reduced M4 receptors in the caudate-putamen and hippocampus [70, 71], which may correlate with the presence of both the positive and negative symptoms of schizophrenia. Further, recent studies report that M4 gene receptor polymorphisms are associated with schizophrenia [70, 72], thus further implicating M4 disfunction in the pathogenesis of schizophrenia. A recent study found that M4 agonism cannot inhibit dopamine release without coactivation of the mGlu1 receptor; [73] this finding is supported by data indicating that loss of function mutations in the gene encoding mGlu1 receptor are associated with schizophrenia [74, 75]. Thus, mutations in genes encoding both M4 and mGlu1could increase the risk of schizophrenia through the same pathway.

Medications that augment the M4 receptor have shown efficacy as antipsychotics, likely by downregulating dopamine release. VU0152100, an M4 positive allosteric modulator, has been shown to treat both psychotic symptoms and cognitive disturbances in rodent models of schizophrenia [76]. The same study also revealed that VU0152100, which acts at only M4, was able to reverse amphetamine-induced cognitive deficits, but did not improve cognition from baseline. A very recent phase I study in schizophrenia patients [77], tested the M4 allosteric modulator emraclidine, reporting suitable tolerability across a wide dose range. This study is being followed with placebo-controlled efficacy studies targeting psychotic symptoms with a monotherapy approach [78].

Xanomeline, a muscarinic agonist, is relatively selective for M1 and M4 receptors. Xanomeline has been shown to increase dopamine release in the medial prefrontal cortex [79], where dopamine release is typically lessened in patients with schizophrenia, which may be implicated in the origin of negative symptomology [80]. Xanomeline has also been shown to decrease dopamine release in the midbrain [30], where hyperdopaminergic activity has been correlated with schizophrenia [81]. A 2004 rodent student found that M4-knockout mice have decreased acetylcholine efflux and increased dopamine efflux in the midbrain, thus causing a hyperexcitable dopaminergic state [67]. In turn, by acting as an agonist at M4, xanomeline may be able to increase acetylcholine influx and decrease dopamine efflux in the midbrain, thus causing dopamine to remain intracellularly with reduced availability for neurotransmission. The mechanism by which xanomeline may increase dopamine release in the medial prefrontal cortex is less clear.

### M1 receptors and cognition

Some studies have reported that patients with schizophrenia have reduced levels of M1 receptors in the dorsolateral prefrontal cortex [82, 83]. Other studies have also shown M1 receptor density in the striatum and hippocampus of patients with schizophrenia to be comparable to that of healthy controls [84] and postmortem studies have generally found that levels of cholinergic markers are within normal limits, even in very old samples [85]. When an M1 receptor (a G-protein coupled receptor) is activated by acetylcholine, phospholipase C is activated by the associated G-protein and intracellular calcium stores are released [86]. M1 has been primarily implicated in cognitive processes, as M1 receptors are able to potentiate N-methyl-D-aspartate (NMDA) signaling [87, 88], which is critical to synaptic plasticity and, accordingly, learning and memory. M1 knockout mice display decreased cognitive function and increased amyloid formation [89–92], suggesting that M1 dysfunction may play a two-factor role in cognitive decline in AD. Studies conducted in animal models have shown that M1 activation can mediate amyloid processing, thus decreasing amyloid formation and tau hyperphosphorylation [93, 94]. These findings suggest that agents acting at the M1 receptor may be able to act in a disease-modifying role for dementia processes.

The cholinergic hypothesis of AD states that loss of memory function occurs as the result of a progressive decrease in density of basal forebrain cholinergic neurons to the hippocampus and cortex [95]. Accordingly, acetylcholinesterase inhibitors, which increase the availability of acetylcholine in synapses, are used to alleviate symptoms in the dementia phase of AD by augmenting cholinergic function [96]. Additionally, several M1 agonists have been successful in increasing cognition in AD patients [97], but usage has been limited by concomitant activation of M2 and M3 receptors resulting in intolerable side effects or by the need to administer medication intravenously (e.g., physostigmine).

Interestingly, while acetylcholinesterase inhibitors can increase cognition and memory modestly in AD, they have been consistently unsuccessful in treating schizophrenia-related cognitive impairment [98, 99]. However, drugs that agonize M1, such as xanomeline, have yielded improvements in cognition in rodent models of schizophrenia [100] and in recent human studies [101]. This discrepancy may suggest minimal alterations in sensitivity of M1 cholinergic receptors in patients with schizophrenia, as increasing availability of acetylcholine in the synapse does not improve cognition but stimulating the M1 receptor through agonist activity (or potentially allosteric modulation) appears to have benefits. The findings regarding normal levels of M1 receptor density in schizophrenia may suggest that M1 agonists may improve cognitive functioning even if the origin of cognitive dysfunction is not due to pathological changes in M1 receptors. A parallel to this possibility is the findings studies of stimulant treatment in schizophrenia, whereby treatment with amphetamines improves cognition and response to cognitive training even in the presence of dopamine D2 blockade.

Alternative strategies for M1 regulation include allosteric modulators. Such compounds and strategies have been shown to reverse memory loss and slow progression in mouse models of prion disease [102]. These compounds have been reported to have reduced side effect potential, particularly peripheral effects, compared to M1 agonists [103]. Thus, this strategy has the potential for cognitive enhancement with reduced off-target side effects [104].

### Peripheral effects of muscarinic agonism

While M1 is preferentially expressed in the CNS, it is also present in gastric and salivary glands and enteric ganglia [105]. Accordingly, while M1 agonism can improve cognition, it can also cause peripheral side effects such as increased secretions and increased gastric motility. In a randomized control trial of xanomeline in AD patients [23], more than half of patients receiving high-dose xanomeline (225 mg per day) discontinued use due to side effects, including excessive sweating, nausea, vomiting, increased salivation, diarrhea, and fecal incontinence. Of particular concern, 12.6% of the high-dose patients suffered syncope while taking the medication.

While xanomeline has been shown to be functionally selective for M1 and M4 receptors, it is capable of binding to all muscarinic receptors, including M2 and M3 [106, 107]. M2 is largely expressed in the heart and smooth muscle, and M2 agonism can result in bradycardia and increased gastrointestinal motility [108]. Similarly, M3 is widely expressed in smooth muscle and glands; M3 agonism can result in increased salivation, urinary urgency, and incontinence, and increased gastrointestinal motility [109]. As discussed above, M1, while preferentially expressed in the CNS, is also present in salivary glands and the enteric ganglion. As xanomeline functionally selects for M1 and M4 but may have some off-target activity at M2 and M3, it is plausible that the intolerable side effects of xanomeline are primarily caused by peripheral M1 agonism in the salivary glands and enteric ganglia but may also result from lesser agonism of M2 and M3.

### Xanomeline-trospium

Trospium chloride is a pan-muscarinic antagonist; its structure contains a tertiary amine, rendering it highly polar and unable to cross the blood brain barrier and enter the CNS [110]. It is approved for used for the treatment of overactive bladder. In a new formulation, xanomeline is combined with trospium with the intent of blocking xanomeline’s peripheral agonism to minimize side effects. A recent phase 2 trial of xanomeline-trospium found that, by adding trospium, the side effects of xanomeline are drastically reduced, with 20% of patients receiving xanomeline-trospium discontinuing the drug compared to 21% of patients receiving placebo [37]. 54% of xanomeline-trospium patients reported adverse events, as compared with 43% of placebo patients. Interestingly, two of the most reported adverse events in the xanomeline-trospium group were constipation and dry mouth, which is suggestive of a highly effective peripheral muscarinic blockade, as the side effects of xanomeline alone include diarrhea and excessive salivation. Importantly, while the earlier trial of xanomeline alone in patients with AD reported a high incidence of syncope [23], there were no episodes of syncope reported in patients taking xanomeline-trospium.

While combining xanomeline with trospium appears to lower rates of cholinergic side effects, xanomeline remains effective for schizophrenia in this combination. In the same phase 2 trial, patients taking xanomeline-trospium received a significant benefit over placebo on scales evaluating both positive and negative symptomatology. Patients in the xanomeline-trospium group also showed significant benefit over placebo on the Clinical Global Impression-Severity scale (CGI-S) [111], which is intended to serve as a measure of overall clinical status. Accordingly, xanomeline-trospium shows potential as a novel treatment for schizophrenia with a greatly improved side effect profile over xanomeline alone.

Importantly for the arguments presented in this paper, a recent publication based on data from the same phase 2 study reported statistically significant cognitive benefits from xanomeline-trospium compared to placebo [101]. After excluding participants who had invalid baseline scores on the computerized cognitive battery, participants receiving active treatment improved by approximately 0.3 SD more than the participants receiving placebo. This is a level of improvement consistent with that seen in previous phase 2 studies of other cognitive enhancing agents. Importantly, when participants whose cognitive performance was in the normal range were censored from the analysis, the separation between active and placebo treatment increased to 0.71 SD.

### Increasing interest in adverse impact of anticholinergic medications?

Several very recent studies have revisited the impact of anticholinergic burden with a focus on serious mental illness. For instance, Joshi et al. [112] reported on 1120 participants with schizophrenia or schizoaffective disorder who participated in a large-scale genomic study, COGS-2 [113]. The authors reported that all cognitive domains were affected by current anticholinergic burden and that demographic factors were not a contributor. In two related studies, Eum et al. [114] reported that adverse impacts of anticholinergic burden were preferentially detected in participants with schizophrenia, and not in schizoaffective or bipolar participants. In a GWAS follow-up of this sample [115], the authors reported that they identified five variants that were significantly associated with global cognitive performance, with these variants located at the chromosome 3p21.1 locus, with the top SNP in the inter-alpha-trypsin inhibitor heavy chain 1 (ITIH1) gene (*P* = 3.25 × E-9). The inclusion of anticholinergic burden as a predictor improved association models between the loci and cognitive performance (*P* < 0.001) and additional significant SNPs were identified. This genomic locus (3p21.1) was previously reported to be associated with both risk for psychosis and with cognitive performance in the general population [see Davies et al., 17]. Further, this association was replicated by the same investigators in a largely treatment naïve first episode sample, suggesting that the findings were not related to prior medication exposure. Later research could easily be directed at genomic correlates of adverse response to anticholinergics examining potentially moderating effects of genomic factors with greater adverse impacts of anticholinergics.

### Other potential uses for muscarinic agonists: broader applicability?

Given the findings that M1 and M4 muscarinic receptors have different functions, treatments that target these receptors may have several different uses. While previous studies have shown that xanomeline had efficacy for cognition and psychosis in both AD and schizophrenia, there are other conditions that share cognitive impairments. Further, for individuals whose psychotic symptoms are refractory to antipsychotic treatments, alternative strategies for down-regulation of dopamine activity could be considered as an intervention. These uses can include cognitive targets, as well as symptomatic targets that may be related to excessive striatal dopaminergic D_2_ activity. Although there are clear interactions with muscarinic agonists and neurotransmitter systems implicated more clearly in schizophrenia than other conditions (e.g., NMDA, D2), the impacts of these compounds may be more broadly applicable, even in the absence of a very strong signal for abnormal activity of dopamine or glutamate.

### Age-related cognitive changes

It has been found since the advent of standardized cognitive testing that there are normative age-related changes in cognitive functioning. These changes are greatest for what are referred to as fluid cognitive abilities, including the ability to process novel information, solve new problems, as well as concentration, attention, and processing speed [116]. Even in individuals with no evidence of gross changes in cognitive performance, some abilities are performed at levels 30–50% less efficient in the ninth decade of life than in the third. These normative differences are larger in individuals with lower levels of performance, such that individuals whose performance at age 25 was at the 50^th^ percentile have relatively greater differences in their performance on indices of fluid cognition across the lifespan than individuals with higher levels of performance.

Crystallized knowledge includes vocabulary and other fact-based information. These indices do not change with age and may increase slightly over time within individuals [117]. Thus, the situation where individuals notice that they do not seem as “sharp” with aging is associated with objectively reduced performance in certain domains compared to earlier years and an increased risk of non-normative cognitive decline even in the absence of a diagnosable condition [118]. The data reviewed above regarding the potential efficacy of M1 agents for treatment of cognitive impairment in AD would likely also be applicable to the treatment of mild cognitive impairment (MCI), which in many cases is an early stage of AD. The advent of PET scan-based and blood-based AD biomarkers has suggested that MCI is commonly associated with amyloid deposition and this deposition is a risk factor for increased conversion to dementia [119]. Further, reductions in M1 activity are also shown to correlate with increased deposition of amyloid [120], suggesting that early treatment with M1 agonists may have the potential to improve cognitive functioning and to have a potentially disease modifying influence.

A subset of aging individuals reports more substantial cognitive declines, referred to as “subjective cognitive declines”. The presence of subjective memory complaints, even in the context of unimpaired performance on traditional screening measures, has been reported to be correlated with development of dementia at a 3-year follow-up interval [121]. In that study, participants with memory complaints and unimpaired Mini-Mental State Exam (MMSE) scores were 2.7 times as likely to be diagnosed with AD at the follow-up compared to the unimpaired cases without memory complaints. These individuals can be detected with validated rating scales, such as the Alzheimer’s Disease Cooperative Study Prevention Instrument Project – Mail-In Cognitive Function Screening Instrument (ADCS-MCFSI). This rating scale assesses subjective cognitive decline across domains that are relevant to the early stages of MCI. Recent studies have suggested that individuals identified on this rating scale as having definite subjective cognitive decline were more impaired on an array of cognitive measures and performance assessments of everyday functional skills [122].

The subjective cognitive decline population has been targeted for pharmacological and cognitive training interventions. For instance, a recent trial randomized participants with subjective cognitive decline (limited to 1.0 SD below normative standards on structured testing) to computerized cognitive training combined with either the novel antidepressant medication vortioxetine or placebo [123]. The authors reported that the combined therapy was more effective at improving targeted assessment of fluid cognition than monotherapy with cognitive training. As the objective performance changes seen in subjective cognitive decline are consistent with both the early signs of mild cognitive impairment and are responsive to treatment with plasticity-focused interventions, these conditions might be an additional target for treatment with M1 agonists. A further use of these M1 agonists might be as a safer strategy for pharmacologically augmented cognitive training (PACT [124]) than the stimulant strategies described above for schizophrenia.

### Partial treatment response in schizophrenia

Although many patients with schizophrenia have a good response to antipsychotic treatment and achieve remission, there are a proportion of cases who manifest a partial or negligible response even with confirmed adherence [125]. The presence of residual symptoms is commonplace, even when clozapine is prescribed; these cases are designated as ultra-treatment resistant [126]. Even in cases who are adherent to long-acting antipsychotic treatment, rates of partial response (defined as failure to achieve remission) can approximate 60% [127]. Further, relapse can occur even during confirmed continuous treatment with long-acting injectable medications, with a recent study suggesting a relapse rate slightly higher than 20% per year [128]. The relapse rate was only 15% in cases who had achieved full remission.

These data suggest a need to develop additional strategies to address failures to achieve or sustain remission in schizophrenia. The data reviewed above regarding direct downregulation of dopaminergic activity in critical subcortical areas suggests that M4 agonists may have the potential to provide an additive benefit in terms of dopamine regulation compared to D2 antagonists. Further, the finding that dopamine release in the cortex in critical regions relevant to cognition is possibly even upregulated by M4 agonists suggests that this may be an additional beneficial strategy. Other compounds can downregulate dopamine activity without direct dopamine antagonism, such as trace amine-associated receptor 1 (TAAR1) antagonists that also appear to operate by downregulating dopaminergic activity in critical brain regions, such as the ventral tegmental area [129]. Alternative strategies to downregulate dopaminergic activity without D2 antagonism may also reduce risks for side effects such as extrapyramidal symptoms (EPS) and tardive dyskinesia (TD) while offering incremental benefits for control of psychotic symptoms.

## Conclusions

In this paper we argue that there are alternatives to attempts to reduce cognitive deficits through development of disease specific pharmacological interventions. Cognitive functioning in serious mental illness has considerable cross-diagnostic overlap and considerable genomic overlap with cognition, intelligence, and educational attainment in the general population. Thus, identification of pharmacological targets that have a broad influence on cognition action across the general and pathological populations appears to be a viable strategy. The muscarinic cholinergic system appears to be one such candidate. This system has attracted attention for its role in aging related cognitive changes, in the development of dementia, and in schizophrenia. The muscarinic system interacts with several different transmitters that are also affected in schizophrenia, including dopamine and glutamate, but its importance clearly transcends a single condition.

Previous intervention attempts targeting this system were handicapped by fact that peripheral activity of acetylcholine includes gastric and cardiac functions which were notably impacted by M1 agonists with both peripheral and central effects. New developments in medicinal chemistry have led to the development of a compound with an anticholinergic compound that does not enter the CNS and preliminary results suggest increased safety. Further developments include focusing on the development of positive allosteric modulators that may have reduced off target effects in the periphery. The muscarinic system also regulates dopamine at certain receptor subtypes and compounds are in development to target both M1 and M4 receptors to improve cognition and reduce dopamine activity respectably.

Targeting the muscarinic system has some parallels to the use of stimulant medication, which has also been shown to have cross-diagnostic benefits on cognition as well as potential to augment the effects of other training interventions. It is possible that muscarinic treatments directly targeting cognition and or used as an adjunct to other interventions could have improved safety potential compared to amphetamine and related treatments. Further, the broad age range of cholinergic effects on cognition and the fact that fluid cognition commonly changes for the worse with aging suggests intervention potential that is broader than would be present with stimulant medications.

The eventual application of muscarinic strategies will be dependent on upcoming information on safety and tolerability over longer time periods. Recent conference presentations have suggested similar tolerability and efficacy in a large-scale phase 3 study, although those data have not been reviewed by the FDA or published in a peer-reviewed journal [130]. Pending that information, it seems plausible that muscarinic agonist therapy has broad applicability for use in a wide range of potential uses.
